# Early Classification of Pathological Heartbeats on Wireless Body Sensor Nodes

**DOI:** 10.3390/s141222532

**Published:** 2014-11-27

**Authors:** Rubén Braojos, Ivan Beretta, Giovanni Ansaloni, David Atienza

**Affiliations:** Embedded Systems Laboratory, École Polytechnique Fédérale de Lausanne, 1007 Lausanne, Switzerland; E-Mails: ivan.beretta@epfl.ch (I.B.); giovanni.ansaloni@epfl.c (G.A.); david.atienza@epfl.ch (D.A.)

**Keywords:** embedded signal processing, wireless body sensor nodes, electrocardiogram, classification

## Abstract

Smart Wireless Body Sensor Nodes (WBSNs) are a novel class of unobtrusive, battery-powered devices allowing the continuous monitoring and real-time interpretation of a subject's bio-signals, such as the electrocardiogram (ECG). These low-power platforms, while able to perform advanced signal processing to extract information on heart conditions, are usually constrained in terms of computational power and transmission bandwidth. It is therefore essential to identify in the early stages which parts of an ECG are critical for the diagnosis and, only in these cases, activate on demand more detailed and computationally intensive analysis algorithms. In this work, we present a comprehensive framework for real-time automatic classification of normal and abnormal heartbeats, targeting embedded and resource-constrained WBSNs. In particular, we provide a comparative analysis of different strategies to reduce the heartbeat representation dimensionality, and therefore the required computational effort. We then combine these techniques with a neuro-fuzzy classification strategy, which effectively discerns normal and pathological heartbeats with a minimal run time and memory overhead. We prove that, by performing a detailed analysis only on the heartbeats that our classifier identifies as abnormal, a WBSN system can drastically reduce its overall energy consumption. Finally, we assess the choice of neuro-fuzzy classification by comparing its performance and workload with respect to other state-of-the-art strategies. Experimental results using the MIT-BIH Arrhythmia database show energy savings of as much as 60% in the signal processing stage, and 63% in the subsequent wireless transmission, when a neuro-fuzzy classification structure is employed, coupled with a dimensionality reduction technique based on random projections.

## 1. Introduction

Ongoing changes in world demographics and the prevalence of unhealthy lifestyles are imposing a paradigm shift in the healthcare landscape. Nowadays, chronic diseases, and in particular cardiovascular disorders, represent the most common cause of death [1]. Continuous monitoring, needed for the supervision of patients affected by a cardiopathy, strains the resources of healthcare systems. Technologies based on Wireless Body Sensor Nodes (WBSNs) effectively alleviate this burden, allowing for long term and autonomous recording of biological signals, even outside a hospital environment.

WBSNs are miniaturized, wearable embedded devices able to acquire and wirelessly transmit biological data. A major field of application of WBSNs is the ambulatory acquisition of electrocardiograms (ECGs), which represent the electrical activity of the heart. Body sensor nodes allow long term monitoring of subjects, while producing little discomfort and requiring minimal medical supervision.

A recent trend in WBSNs, driven by the progress in semiconductor technologies, has been the emergence of *smart* WBSNs [2]. These devices are not limited to acquisition and transmission. On the contrary, they are able to host computationally intensive tasks, allowing the on-node interpretation of sampled data.

In the field of clinical electrocardiography, an important application of smart wireless nodes consists in separating normal and pathological heartbeats, performing an early diagnosis step. For this task, many off-line algorithms have been proposed in the literature based on the morphology of the heartbeat [3–5]. However, their real-time implementation on embedded platforms poses considerable challenges due to their high computational requirements.

In this paper, we propose a framework to design real-time, lightweight heartbeat classifiers based on a neuro-fuzzy structure, detailing the required optimizations to efficiently execute them on a WBSN. The framework extends and completes our previous work on neuro-fuzzy classification of heartbeats [6], by introducing the following novel contributions:

- We propose and compare the effectiveness of different dimensionality reduction strategies, which are coupled with the embedded classifier to reduce the computational complexity. The proposed strategies include random projections, principal component analysis, automated fiducial points detection, and proper combinations of the three.
- We quantitatively assess, from a performance and workload standpoint, the effectiveness of neuro-fuzzy classification when compared to popular alternative techniques, namely, support vector machines and linear discriminants.
- We validate the accuracy of the classification framework, and measure the run-time performance of the implemented application on a real-world WBSN [7]. Tests are conducted considering heartbeats from MIT-BIH Arrhythmia database [8] with three different morphologies: normal sinus rhythms, left bundle branch blocks and premature ventricular contractions.

Experimental results highlight that a solution based on random projections and an optimized neuro-fuzzy classification scheme can identify more than 95% of abnormal heartbeats, while using a small fraction of the available SoC memory and computing resources.

The remaining of the paper is structured as follows: Section 2 presents the goal of this work, its motivation and main challenges. Section 3 acknowledges related efforts in the field of heartbeat classification, Section 4 describes the implementation of the classification application, detailing the optimization and training steps performed to meet the WBSN constraints with a negligible loss of accuracy. The experimental setup is discussed in Section 5, and a comparative assessment of the performance of different classifications is reported in Section 6. Finally, Section 7 concludes the paper.

## 2. Motivation, Target Application and Proposed Approach

**Figure 1.**
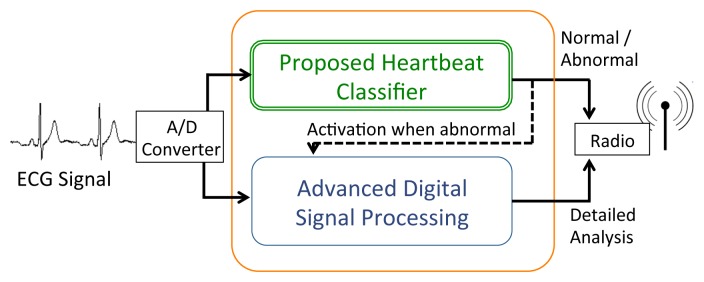
Target system featuring a classification block that selectively activates a detailed digital signal processing chain.

Early classification of heartbeats has potential benefits both in the clinical practice and in the design of WBSNs. On the diagnostic side, it can provide helpful information for speeding up the visual inspections of lengthy ECG recordings by the medical staff, who can focus only on those beats presenting pathological characteristics. From the perspective of system design, the advantages are two-fold: first, if a detailed diagnosis is performed off-node, it can be desirable to transmit or store only pathological beats on the WBSN, thus greatly reducing either the energy employed for wireless transmission or the data storage requirements, respectively. Second, if the detailed analysis of heartbeats is executed on the WBSN, computation effort can be reduced by activating these advanced algorithms only when abnormal beats are detected, thus drastically decreasing the computational requirements and therefore the power consumption. This last scenario (depicted in Figure 1) is the target of this paper. By decoupling early and detailed analysis, and performing the latter only on a small fraction of the acquired bio-signal, our work aims to maximize the energy efficiency of autonomous devices for personal health monitoring. Without loss of generality, we herein focus on a context where pathological heartbeats occur less frequently than normal ones, which is the usual case in long-term ECGs acquisitions.

Up to date, classification of pathological heartbeats has been tackled using off-line algorithms. However, the implementation of these algorithms on WBSNs represents an important challenge due to the high run-time demands. In a previous work [6], we have preliminarily shown how *neuro-fuzzy classifiers* (NFCs) [9] can be optimized for and implemented on the constrained resources typically available on WBSNs, while still providing high classification accuracy and meeting real-time constraints. In this paper, we further investigate the potential of neuro-fuzzy classification, by providing a quantitative comparison with respect to other strategies compatible with embedded execution: support vector machines [3] and linear discriminants [10].

Another important aspect to consider when devising on-node classification is the high dimensionality of the heartbeat representation. Using standard off-line techniques, tens of samples before and after the center peak of the heartbeat are required to perform a reliable classification. To reduce this amount of data and cope with the limited resources of WBSNs, in this paper we propose and comparatively evaluate different approaches allowing a compact representation of heartbeats: *Random Projections* (RPs) [11], Principal Component Analysis (PCA) [12] and automated Fiducial Points Detection (FPD) [13].

## 3. Related Work

As opposed to the existing approaches for off-line classification of heartbeats based on the morphology analysis ([3–5]), on-node classification has to cope with the limited computation resources that are available on a WBSN while providing a comparable accuracy.

Among the classification techniques that could be adapted to on-node execution, neuro-fuzzy classifiers (NFCs) [9] represent a promising option. Their ability to explicitly express uncertainty in classification, given by the employed *fuzzy values*, makes them particularly well-suited to the problem of heartbeat classification, as shown in [14,15]. NFCs can be effectively trained using established methods, the most common being the gradient descent algorithm described in [15] and the scale conjugate gradient introduced in [16,17], which is employed in this work. This classification method is computationally simpler and presents lower memory requirements than other existing techniques such as gaussian Support Vector Machines (SVMs) [3], while being more accurate than simpler solutions based on linear SVMs and Linear Discriminant Analysis (LDA) [10]. Consequently, as highlighted by the experimental evidence presented in Section 6.4, optimized NFCs are a suitable candidate for execution in embedded WBSNs.

Several state-of-the-art strategies for off-line classification of ECGs can be found in the literature. They can be distinguished based on the methodology employed to extract the features of individual heartbeats, which later are the input of the classifier. A first methodology considers the extraction of the most important component in the ECG signal in an appropriate subspace, employing either independent or principal component analysis (ICA [18] or PCA [4], respectively). A different approach, introduced in [19,20], relies instead on the detection of morphological features, such as the presence, duration and shape of the heartbeat characteristic waves. A third methodology focuses on (possibly trained) random linear combinations of the input samples, employing Random Projections (RP) [21,22] for representing heartbeats with a few coefficients. In particular, *Achlioptas* projections [11] adopt matrices consisting only of the elements 0, 1 and −1, thus allowing a compact representation of the projection matrix and requiring low run-time resources.

However, all the aforementioned works either only target off-line analysis of ECG heartbeats or, in the case of [20], are not focused on minimizing the computational workload of the proposed classification methodology when it is executed on a wearable device. Instead, we herein investigate how heartbeat classification can be effectively realized directly on an embedded platform with tight run-time and memory constraints. To achieve this, we propose a framework that combines a state-of-the-art lightweight classifier with multiple dimensionality reduction techniques, providing experimental evidence on classification performance as well as resource requirements on wearable platforms.

## 4. Classification Methodology

**Figure 2.**
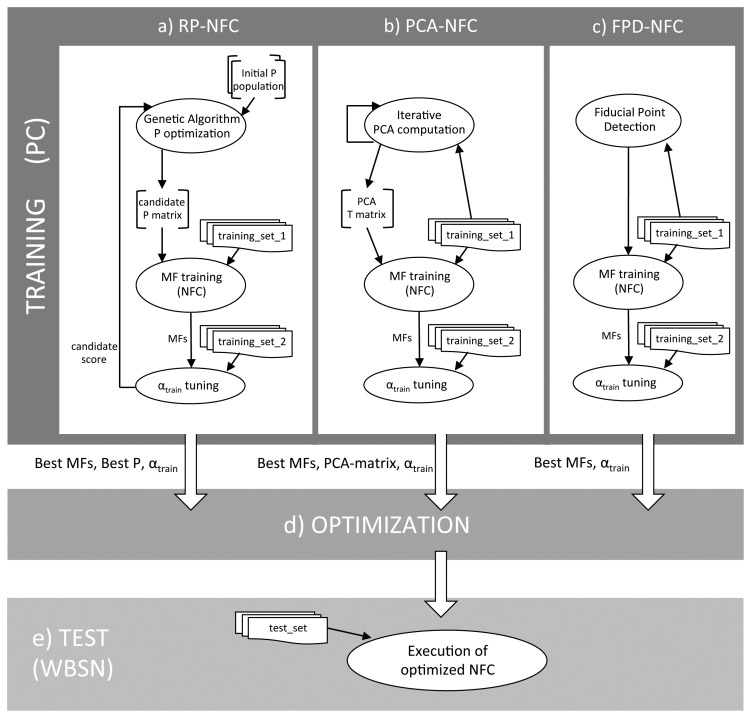
Classification framework: *Top (PC-side):* NFC training using Random Projections (**a**); Principal Component Analysis (**b**) and Fiducial Points Detection (**c**); Optimization for WBSNs (**d**); *Boottom (WBSN side):* Real time execution of the optimized implementation (**e**).

The high-level scheme of the proposed framework for on-node early classification of normal and pathological heartbeats is shown in Figure 2. The framework can be divided into an off-line training phase (Figure 2, top), in which the parameters of both the classifier and the dimensionality reduction technique are derived, and a test phase (Figure 2, bottom), discerning normal and pathological heartbeats at run-time on the WBSN.

Different dimensionality reduction strategies require a different training approach, as illustrated in Figure 2a–c. In the case of PCA (Figure 2b), principal components are derived from an initial set of heartbeats before the neuro-fuzzy classifier is trained. Conversely, when random projections are used (Figure 2a), a concurrent optimization of the NFC classifier and of the random projection matrix is required. Finally, fiducial points detection does not require any specific optimization, as they are extracted from the heartbeats by means of a delineation algorithm, hence in this case only the NFC has to be trained (Figure 2c).

The training and test phases of the classification framework have different constraints. On the one hand, the training phase is performed off-line on a host workstation, which employs high-precision floating-point data representation in order to obtain an accurate framework set-up. On the other hand, the test phase and the actual classifier is eventually executed on an embedded WBSN, being therefore tightly constrained in terms of memory footprint and computation resources, since only integer arithmetic is admitted and no exponential operations are possible. As a consequence, it is mandatory to transform the classifier after the training (Figure 2d) to lower its computational requirements according to the embedded platform capabilities. Our proposed methodology to realize this step is detailed in Section 4.3, while Sections 4.1 and 4.2 describe the training of the dimensionality reduction techniques and of the NFC, respectively.

### 4.1. Dimensionality Reduction Approaches

Reducing the dimensionality of the heartbeat is an effective technique to simplify the complexity of the classification problem. We explore three different solutions to achieve this objective, which result in three distinct training procedures (Figure 2a-c) that are described in the following paragraphs.

#### Random Projections (RPs)

Random projections allow to represent the ECG by means of a low number of coefficients, which are obtained by multiplying the input vector of samples by a random projection matrix. In order to improve the run-time performance of the classification, we require the RP matrix to be sparse. This requirement is fulfilled by a *k* × *d* Achlioptas matrix (**P**) [11], where *d* is the number of digital samples acquired for each heartbeat and *k* is the number of desired coefficients in the random projection, with *k* ≪ *d*. The elements of **P** are defined as:

$${\text{P}}_{k,d}=\{\begin{array}{cc} +1 & \text{with probability}\frac{1}{6}\\ -1 & \text{with probability}\frac{1}{6}\\ 0 & \text{with probability}\frac{2}{3} \end{array}$$

The dimensionality reduction is then achieved by randomly-project the vector *v* according to the following equation: *u_RP_* = **P***v*. Because of the structure of the Achlioptas matrix, each row of **P** indicates which elements of *v* have to be added (possibly negated) to derive the corresponding vector *u_RP_*, without using an actual multiplication.

Even though the approximation error introduced by random projections is theoretically bounded [21], in practice we observed that certain RP matrices perform better than others. As a consequence, the generation of **P** requires a training process (Figure 2a). The aim of the training is to derive a matrix **P** which leads to a high-quality classification, resulting in a joint optimization process of the RP matrix and of the NFC. In the proposed approach, this is achieved by means of a genetic algorithm [23]. The algorithm starts from an initial population of random matrices and, for each of them, tunes the corresponding NFC (as described in Section 4.2) over a set of projected heartbeats (*training_set_1*). Each of the obtained RP-NFC pairs is then evaluated over a different and larger set of heartbeats (*training_set_2*). According to the result of the evaluation, the genetic algorithm selects the proper chromosomes (*i.e.*, the best **P** matrices) and performs mutation and crossover over them to refine the random projection. According to our experiments, an initial population of 20 randomly-generated matrices, and an exploration of 30 generations by the genetic algorithm, are sufficient to converge to a close-to-optimal **P**.

#### Principal Component Analysis (PCA)

Principal component analysis retrieves the set of linear projections on a set of orthonormal axis, where the variance in the input data is maximized [4]. The columns of the PCA projection matrix **T** of size *k* × *d*, with *k* ≪ *d*, are the *k* leading eigenvectors of the covariance matrix of the input data, which is defined as:

$$\text{S}=1/N\sum_{i=1}^{N}{\left(v_{i}-\text{E}\left[v\right]\right)}^{T}$$

where *N* is the number of elements in the *training_set_1* dataset, *v_i_* is the *i*-th element of the vector containing the digital samples of a heartbeat, and E[*v* ] is their mean value. As in the case of RPs, dimensionality reduction is performed by a matrix-by-vector multiplication: *u_PCA_* = **T***v*.

The extraction of the PCA matrix **T** only depends on the algebraic properties of the input vector *v* and on the numbers of projection axis *k*, therefore it can be performed independently from the NFC classifier training (Figure 2b). In particular, herein the PCA matrix is derived according to the iterative solution presented in [24]. On the other hand, as opposed to the RP case, **T** is not sparse in general, leading to a more complex run-time implementation that also involves multiplications to compute *u_PCA_*.

#### Fiducial Points Detection (FPD)

**Figure 3.**
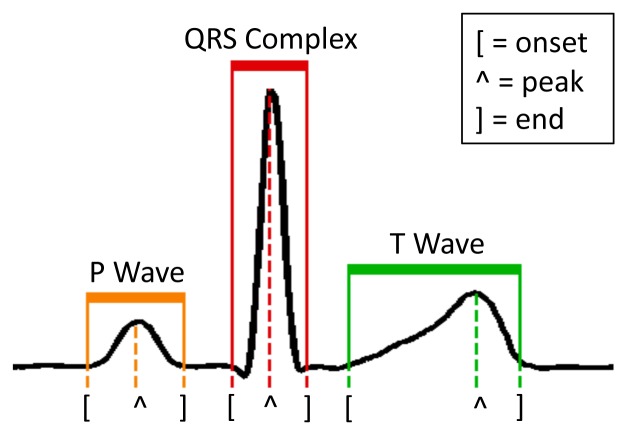
Delineated normal heartbeat [25].

Differently from RPs and PCA, the detection of morphological features explicitly *interprets* the input ECG signal. In particular, FPD aims at retrieving the position of the fiducial points (onset, peak and end of the P and T waves, and the beginning and end of the QRS complex) of each heartbeat with respect to the position of the R peak (Figure 3). The considered fiducial points (8 in total) constitute the coefficients of the vector *u_FPD_*, which is the dimensionally-reduced representation of *v*, and which is used to subsequently feed the NFC classifier.

In the proposed framework (Figure 2c), we perform FPD using the lightweight algorithm proposed in [2]. The algorithm is based on the digital wavelet transform (DWT) decomposition, which transforms each characteristic ECG wave into tuples of maxima and minima in the DWT domain. Since the different waves present distinct frequency contents, their fiducial points are retrieved at different scales, the QRS complex having a stronger component at lower scales than the P and T waves. The DWT delineation shows good run-time properties that make it suitable for on-node fiducial point extraction, and its robustness makes it a good choice to deal with pathological beats with abnormal morphologies.

### 4.2. Neuro-Fuzzy Classifier Training

**Figure 4.**
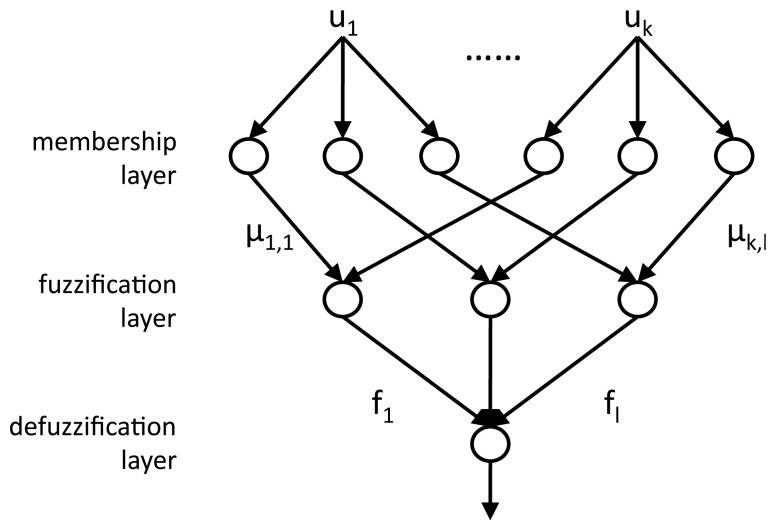
Three-layer neuro-fuzzy classifier.

The coefficients *u*, representing the dimensionally-reduced heartbeat, are the inputs to the multi-layered NFC, whose structure is illustrated in Figure 4. A first *membership* layer employs Membership Functions (MFs) to compute, for each coefficient *u_k_*, a membership grade *μ_k,l_* for each of the three target classes *l*: normal beats (*N*), left branch blocks (*L*) and premature ventricular contractions (*V*). After the training of the NFC, which is performed using the gradient descent algorithm [15], the obtained MFs are defined as gaussian curves, defined by their center *c* and variance *σ*:

$$\mu_{k,l}\left(u_{k}\right)=\exp\left(\frac{-{\left(u_{k}-c_{k,l}\right)}^{2}}{2\sigma_{k,l}^{2}}\right)$$

where *l* ∈ {*N*, *V*, *L*}.

In the subsequent *fuzzification* layer, the membership grades of all the coefficients for each class are combined by means of a product: $f_{l}=\prod_{k}\mu_{k,l}$. The resulting *fuzzy values* quantify how likely the examined heartbeat belongs to that specific class: the larger the value with respect to the values of the other classes, the higher the confidence in the correctness of the assignment.

The third *defuzzification* layer of the NFC marks each beat as either normal or pathological, by considering the two largest fuzzy values (*M*1*_f_*, *M*2*_f_*) and the sum of all of them $S=\sum_{l}f_{l}$.

If (*M*1*_f_* − *M*2*_f_*) *≥ α_train_* · *S* (with *α_train_* ∈ [0,1]), the beat is assigned to the class with the maximum fuzzy value (*N, V, L*). Otherwise, the beat is marked as *unknown* (*U*). *V, L* and *U* beats are considered as potentially pathological, while *N* beats are marked as normal.

The choice of a proper defuzzification coefficient *α_train_* gives the flexibility to unbalance the classifier training process, *i.e.*, it allows to define an upper bound on the number of abnormal beats that are incorrectly classified as normal. Once this percentage is fixed, the performance metric used to train and score the classifier is then the percentage of normal beats correctly detected, and therefore discarded for detailed analysis.

### 4.3. Resource-Constrained Optimization Phase

The dimensionality reduction technique and the trained classifier cannot be employed *as they are* on a WBSN platform, due to the available limited resources. In this regard, several considerations have to be taken into account. First, data must be represented in the integer domain, as opposed to the floating-point format used in the training phase. Then, the complex MFs employed in the NFC, which require prohibitive exponential operations for embedded platforms, must be simplified. In addition, the NFC fuzzification layer needs be analyzed to prevent overflows when performing the product operation. Finally, special care must be taken regarding the memory required to store tables such as the RP matrix, or to represent the different parameters derived during the training phase. In this section, we detailed the devised strategies performing these steps (Figure 2d), thus enabling the implementation of the proposed classifier on a WBSN.

#### Membership functions linearization

**Figure 5.**
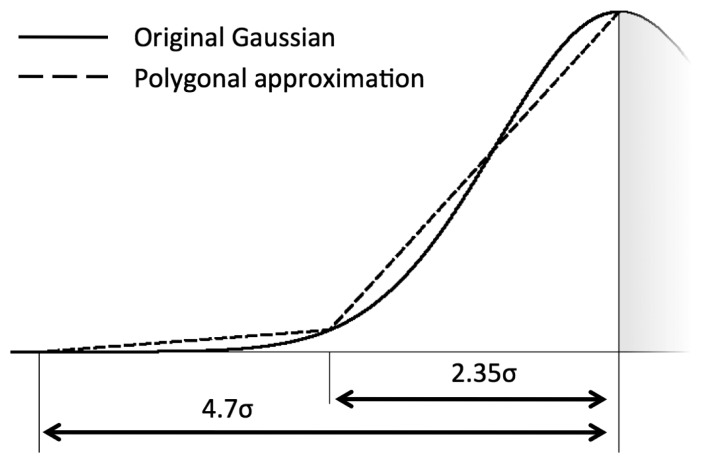
Linear approximation of gaussian MFs in the range [−4.7*σ*, 0], compared to a gaussian curve.

We propose a linear segmentation of each gaussian MF in the classifier, to avoid the computation of exponentials. Given the centre *c* and standard deviation *σ* of a MF, we map it onto the integer range [0,(2^16^ − 1)] (*i.e.*, a 16-bit representation) according to the following scheme:

$$MF_{\text{lin}}\left(x\right)=\{\begin{array}{ll} 0 & \text{if}\;\left|c-x\right|\geq 4S\\ 1 & \text{if}\;4S>\left|c-x\right|\geq 2S\\ \text{lin}.\text{approx}1 & \text{if}\;2S>\left|c-x\right|\geq S\\ \text{lin}.\text{approx}2 & \text{if}\;S>\left|c-x\right| \end{array}$$

where *MF_lin_* is the linearized MF and *S* = 2.35*σ*. The linear approximation segments are graphically represented in Figure 5. As 1 is the smallest non-zero value that can be represented in the chosen integer space, this formulation has the desirable property to be positive in a large range, hence it is rare that a fuzzy value becomes 0 after the defuzzification (product) stage.

#### Fuzzification

In the defuzzification layer, only the ratio between the fuzzy coefficients *f_l_* is relevant, as opposed to their absolute values. The proposed optimization of the fuzzification step stems from this observation, and consists in retaining the maximum precision given the 32-bit representation used for the accumulators of the defuzzification products. In our implementation, the membership grades *μ_k,l_* related to the two first coefficients are multiplied for each of the three classes. The three resulting numbers are left-shifted to the maximum amount so that none of them overflows, and then the least significant 16 bits are discarded. All the subsequent membership grades are then processed in a similar way, thus obtaining the fuzzy values of the beats for the different classes.

#### Defuzzification

The defuzzification layer marks each beat as normal or pathological. The chosen implementation does not employ divisions, and can therefore be efficiently implemented in WBSNs. Moreover, it is possible to choose a defuzzification coefficient *α_test_* different from the *α_train_* that was obtained during the training phase (described in Section 4.2), allowing to adjust the ratio of detected normal and abnormal beats at run-time.

#### Memory-Aware Representations

As mentioned in Sections 3 and 4.1, random projection matrices are composed of only three values (+1, −1 and 0) and are sparse by construction, thus admitting a compact representation where each element can be coded using only two bits. It therefore requires 1/4 of the memory with respect to a corresponding matrix of 8-bits values. On the other hand, a 16-bit are employed for representing the components of the PCA matrices, resulting in a memory footprint that, while still being compatible with a WBSN implementation, is significantly higher with respect to the compact RP case.

## 5. Experimental Setup

To comparatively evaluate the proposed classification strategies using different dimensionality reduction methods, we investigated their performance when identifying the normal (*N*), premature ventricular contraction (*V*) and left bundle branch block (*L*) heartbeats included in the MIT-BIH Arrhythmia Database recordings (available on the Physiobank website [8]). The considered heartbeats were extracted from the MLII lead of each recording. The real-time performance of the proposed classifier is evaluated by means of its actual implementation on a physical embedded platform. In this work, we have employed the state-of-the-art IcyHeart System-on-Chip (SoC) [7], which integrates a low-power microprocessor featuring a clock frequency of 6 MHz and an embedded RAM of 96 KBs.

**Figure 6.**
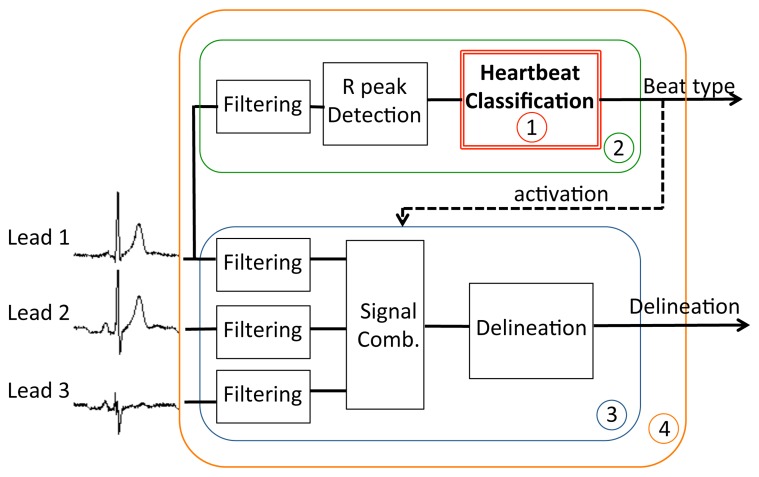
Experimental scenario: classification is used to activate an accurate multi-lead morphological delineation only in case of heartbeat abnormality.

The goal of the classification is to distinguish between normal or pathological beats, in order to trigger a detailed analysis for abnormal beats only, as we mentioned in Section 2. In our case, the general structure shown in Figure 1 is embodied by the system in Figure 6, where the detailed analysis is obtained by a three-lead morphological delineation (MMD) [13,25]. The considered figures of merit of the classifier are the Normal Discard Rate (NDR) and Abnormal Recognition Rate (ARR). The NDR assesses the rate of normal beats that are correctly identified as such with respect to the total number of normal heartbeats, and thus they are discarded for further analysis. Complementarily, ARR reports the percentage of pathological heartbeats that are correctly identified with respect to the total number of abnormal heartbeats. Unless specified otherwise, across the experiments we set a lower bound of 95% on the ARR, therefore the training process will tune the defuzzification coefficient *α_train_* to meet this requirement.

Obtained results are derived from a 4-fold cross-validation process, with heartbeats of the different classes proportionally and randomly divided across folds. Four rounds of experiments have been performed to compute all the results, using a different fold in each round as the test set (*test_set* in Figure 2) and the remaining 3 folds as the training set (*train_set_2* in Figure 2). A random subset of the training set (*train_set_1* in Figure 2) was used to compute the membership functions of the NFC classifier (regardless of the specific dimensionality reduction technique), and to derive the PCA matrix. In *train_set_1*, the classes are equally represented, in order not to overfit the classifier on the largest class (in this case, class *N*). *Train_set_2* is used to adjust *α_train_* during the training and, in the case of using RPs, to drive the genetic algorithm that derives the optimal projection matrix. The composition of the different heartbeat sets is detailed in Table 1.

The ECG recordings in the database are acquired at 360 Hz. We define each heartbeat as the 100 samples preceding the R peak (cf. Figure 3), and the 100 samples that follow it. The peaks are automatically detected using the wavelet-based technique that was firstly proposed in [2].

## 6. Experimental Results

In this section, we demonstrate the effectiveness of the proposed framework in terms of performance and workload. In Section 6.1, we first assess the classification accuracy achieved by coupling the proposed neuro-fuzzy classifier with the different dimensionality reduction techniques presented in Section 4.1. In Section 6.2 we compare their run-time performance on the target wireless node, in terms of execution time and memory requirements. We then prove how the proposed methodology contributes to the overall reduction of the WBSN energy consumption (Section 6.3), using the scenario illustrated in Figure 6 as the reference case study.

Finally, in order to motivate the choice of neuro-fuzzy classification, Section 6.4 provides a quantitative comparison among different alternatives, including support vector machines and linear discriminants.

### 6.1. Comparative Evaluation of the Proposed Dimensionality Reduction Strategies

In this section, we aim to compare the classification accuracy obtained by combining the different dimensionality reduction techniques (RPs, PCA and FPD) with a neuro-fuzzy classifier structure.

When employing RPs and PCA, we considered two different implementations that reduce the dimensionality of the heartbeat to either 8 or 16 coefficients. A larger coefficient set impacts both the size of the RP or PCA matrix and the complexity of the NFC, therefore the decision among the different implementations is a trade-off between classification accuracy and real-time performance (in terms of run-time and required memory). Fiducial points detection (FPD) does not present this flexibility, as each heartbeat is always represented by 8 values, as discussed in Section 4.1: the position of the onset, peak and end points of the P and T waves, plus the onset and the end of the QRS complex, relative to the main R peak (Figure 3). If a point is not detected, its position is assumed to be the one of the detected neighbor fiducial point that is closer to the R peak.

We also explored two combined solutions, in which 8 coefficients derived from projections (either PCA or RP) are concatenated to the 8 detected fiducial points, resulting in 16 input values for the classifier. In these cases, the fiducial points are added after performing dimensionality reduction over the sample vector of the heartbeat and before training the NFC.

In the first set of experiments, we tested all the aforementioned dimensionality reduction techniques, when *α_train_* is trained to obtain a minimum ARR of 95%. The corresponding NDR figures for the different configurations are detailed in Table 2. Three main considerations can be derived from these results. First, results using only FPD are considerably poorer than the ones achieved by the other classification techniques, showing that the information contained in the delineation of the ECG characteristic waves is not sufficient to perform accurate classification. Second, it can be observed that for those methods in which different number of coefficients can be employed (*i.e.*, RP and PCA), the performance does not vary significantly when using a larger dimensionality, as the maximum improvement does not exceed two percentage points. Third, with the given constraint on the minimum ARR, combining RP or PCA with FPD does not improve the NDR. Although this may seem counterintuitive, the reason for this behavior is that including inputs of different nature tends to increase the number of beats that are classified as *unknown* in the defuzzification stage (as defined in Section 4.2). This forces the training process to slightly increase the value of *α_train_* to meet the minimum ARR requirement, at the cost of negatively affect the NDR.

**Figure 7.**
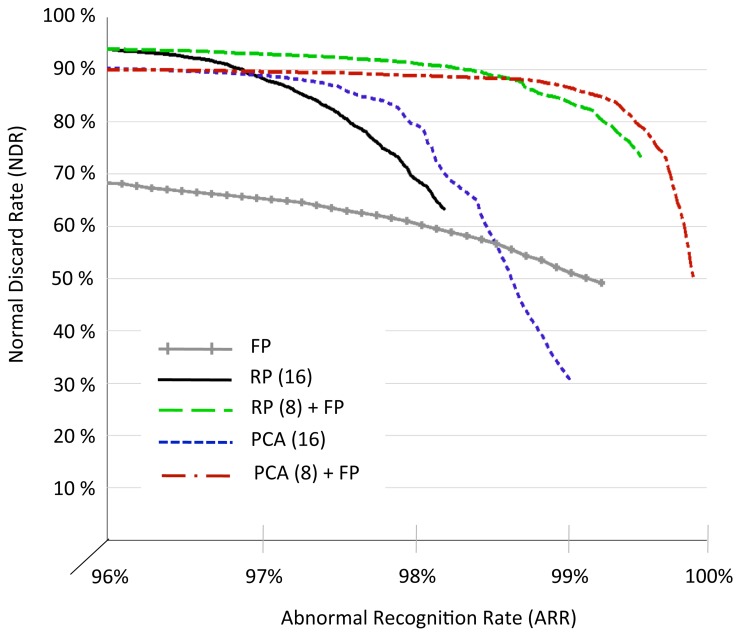
Pareto curves of the NDR/ARR relations for various classification methods obtained by averaging the different values extracted from the 4-fold cross validation process.

In a second set of experiments, we investigated the flexibility of the proposed solutions varying the ARR constraint. In particular, even though *α_train_* is still tuned to get a minimum ARR of 95% on the training set, we scaled the coefficient *α_test_* to obtain different NDR/ARR trade-offs over the 4 rounds of the cross validation process. Figure 7 compares the NDR/ARR Pareto curves obtained for RP (16 coefficients), PCA (16 coefficients), FDP, and the combination of 8 coefficients from RP and PCA with the 8 fiducial points.

Two main conclusions can be derived from the results. On the one hand, when the ARR constraint is set below 97%, RP-based solutions outperform the PCA-based methods reaching an NDR of 94.9% and not requiring the utilization of fiducial points. On the other hand, when the accuracy on the recognition of abnormalities is forced to be closer to 100%, the addition of FPD to the standard RP- and PCA-based methods makes the approach more robust, and leads to high values of NDR and ARR (PCA+FPD being the most reliable alternative). Conversely, as we explained above, using FPD alone for the classification does not provide comparable results.

### 6.2. Run-Time and Memory Size Evaluation

As discussed in Section 2 and shown in Figure 6, the role of the classifier is to activate a detailed analysis for abnormal heartbeats in order to perform selective and computationally intensive advanced processing. In order for the system in Figure 6 to be effective, early detection of pathological heartbeats must not be the computation bottleneck during real-time execution. Therefore, classification should require considerably less effort than performing continuous analysis over the acquired signal.

In this section, we therefore investigate a diagnosis application (system (4) in Figure 6), in which the classification framework is used to trigger the detailed heartbeat analysis. The early heartbeat classification is performed on a single lead (sub-system (2)), whereas the detailed analysis is implemented by a three-lead delineation block (sub-system (3)).

Figure 6 shows that, apart from the classifier itself (sub-system (1)), two additional stages need to be incorporated to complete the proposed one-lead early classification (sub-system (2)). Firstly, a filtering stage is required to remove artifacts and baseline wandering caused by respiration and muscle contractions usually corrupting ECG signals. Secondly, a peak detector has to be employed to identify the heartbeats to classify.

We employed state-of-the-art solutions for the filtering stages, the peak detector and the delineation block, proposed by the authors of [2]. Filtering is performed using morphological operators, a wavelet-based algorithm is used for peak detection and a delineation algorithm using multi-scale morphological derivatives (MMDs) is executed over the root mean square (RMS) combination of the three filtered leads in subsystem (3). Their implementation has been optimized for execution on embedded WBSNs.

Tables 3 and 4 report the computational and memory requirements of the different parts of the considered system depicted in Figure 6, when executed on the IcyHeart WBSN operating at 6 MHz. The first column of the two tables lists the investigated implementations, based on RP, PCA, FPD, and the combined strategies described in Section 6.1. In order to make a fair comparison, experiments have been performed using 16 coefficients as input of the NFC, except in the case of FPD, which only employs 8. The second column reports the experimental results obtained for the classifier block (sub-system 1), while the third one also considers the filtering and peak detection (sub-system (2)). In the fourth column, a system performing three-lead MMD delineation over the full input signal is investigated. This setting reflects the performance of the advanced processing block running continuously, thus analyzing both normal and pathological heartbeats. As it can be seen in the tables, its run-time behavior does not depend on the classification methodology. Finally, in the right-most column of the tables, we provide the values for the complete target system where delineation is performed only on heartbeats marked as abnormal.

By observing the behavior of the classification block, two observations can be extracted. On one hand, the memory footprints of the different investigated methods are different, the RP with 16 coefficients being the least demanding one. On the other hand, the necessary additional computational effort for feature extraction and classification is minimal once the heartbeat is filtered and isolated (less than 1.5% of the duty cycle in all the cases). Additionally, the FPD method benefits from the wavelet decomposition that is already performed during the R peak detection block.

As an assessment of the run-time efficiency of the proposed classification methods, it can be noticed that the main bottlenecks of the classification chain (sub-system (2)) are represented by the input data filtering plus the peak detector, and not by feature extraction and classification. Among the proposed implementations, the RP with 16 coefficients emerges as the best trade-off, providing a comparable performance with the rest of the methods with the lowest memory requirements.

The final, and most important, result is that the duty cycle of the complete system is always substantially lower than an equivalent one that performs a full detailed analysis on all the beats. Analyzing all the beats of the database describe in Table 1 and considering numbers on Table 2, experimental evidence shows that the run-time of sub-system (4), which employs early classification, is 60% lower than the one of sub-system (3), which performs a detailed analysis of each heartbeat, while presenting a small memory overhead (32 kB in the case of RP with 16 coefficients). Moreover, all the dimensionality reduction techniques we proposed achieve savings in terms of duty cycle, even when the classification accuracy is low, *i.e.*, in the case of FPD, showcasing the benefits of early classification.

### 6.3. Improvement of Energy Efficiency

Computation and wireless communication are two major contributors to the power budget of embedded platforms, accounting for approximately 34% of the total energy consumption in a typical WBSN, as shown in [2]. In addition to the reduction in computation requirements discussed in the previous section, the detection of pathological heartbeats contributes to obtain considerable gains in terms of energy efficiency, as the data to be transmitted can be greatly reduced.

In a scenario such as the one illustrated in Figure 6, where the WBSN reports only the R peak of normal heartbeats and all the fiducial points in case of abnormality detection, the usage of the wireless link can be substantially reduced with respect to the case in which all the fiducial points of all the heartbeats are communicated. In the case of the targeted application, where RP with 16 coefficients is used to perform feature extraction before classification, and considering all the heartbeats of the considered database (described in Table 1) as input signals, we achieve a 63% energy consumption reduction in the wireless module. Moreover, the proposed methodology results in a 60% reduction in the required computational effort and, consequently, energy employed for digital signal processing on an embedded microcontroller.

Overall, we achieve an estimated 21% total energy reduction on a typical WBSN execution [2], which includes acquisition, processing and wireless transmission, while still being able to report detailed information related to pathological heartbeats.

### 6.4. Comparison of Classification Methods

The flexibility of the proposed framework allows for different classification strategies to be employed after the dimensionality reduction stage. While the previous sections have focused on a neuro-fuzzy implementation, we herein evaluate the performance and computational cost of multiple popular alternatives, either based on Linear Discriminants Analysis (LDA) or on Support Vector Machines (SVMs) with linear or gaussian kernels.

SVMs separate test data in two classes (here, normal and abnormal heartbeats) through an hyperplane, whose coordinates maximize the separation between instances of the two classes in the training set. A linear SVM assumes that the elements belonging to different classes are linearly separable. Under this condition, classification is performed by pre-computing the separating hyperplane parameters, defined by its normal vector *w* and its offset from the origin *b*. At run-time, an input vector *u* is classified with a simple dot-product operation: *sign*(*w*•*u*+*b*). SVMs can be generalized to non-linear forms by applying the “kernel trick” *i.e.*, mapping the inputs in a high-dimensional space with a suitable non-linear kernel function, such as a gaussian radial function. Run-time classification using non-linear SVMs requires the evaluation of the input elements against a large number of support vectors (SVs), necessitating therefore a much higher workload to evaluate the kernel functions, and a higher memory footprint to store the support vectors. Finally, LDA classifiers utilize a linear combination of the input feature vector, to assign it to one of two classes. Under the assumption that the probability density functions of the classes are normally distributed with identical co-variance, classification is again performed by executing the dot-product of the feature vector with the normal of the separating hyperplane.

The aforementioned classifiers are quantitatively compared in Table 5, which reports the achieved Normal Discard Rates and Abnormal Recognition Rates when performing heartbeat classification. For all the experiments, a 4-fold cross-validation has been performed, and the input feature vector is composed of an 8-coefficient random projection of the signal, plus the fiducial points. A minimum threshold of 95% is originally set on the ARR, and it is progressively reduced with a step of 1% when the training algorithm, based on the scheme depicted in Figure 2a, cannot converge to a solution. Table 5 also summarizes the main operations that are required to perform the classification of a given feature vector.

It can be observed that, on the one hand, linear classifiers (SVM-linear and LDA) have limited workload requirements, but on the other hand they incur in more mis-classifications, showing that the feature vectors are not well separated in a linear space. In particular, none of them is able to meet the minimum threshold of 95% set on the ARR. The SVM-gaussian strategy has better classification performance, but at a price of a memory footprint and a computational effort that is not compatible with a WBSN implementation. In fact, 6KB of memory must be employed only to store the 210 support vectors, an impractical square root operation is required to compute one norm for each SV, and the number of (linearized) gaussian functions for each heartbeat is an order of magnitude higher with respect to a neuro-fuzzy classifier. Experimental evidence suggests that the proposed NFC shows the best tradeoff between accuracy and complexity, achieving similar performance with respect to a SVM-gaussian, while having a small computation and memory overhead with respect to simpler linear strategies.

## 7. Conclusions

In this work, we presented a complete methodology to enable the real-time detection of pathological heartbeats on highly-constrained Wireless Body Sensor Nodes (WBSNs). In particular, we outlined different methods to perform the reduction in the dimensionality of the input data, and we detailed several optimization steps to derive a lightweight implementation of a neuro-fuzzy classifier (NFC). Moreover, we comparatively evaluated the proposed combination with state-of-the-art approaches in terms of accuracy and complexity.

The paper proposes two major contributions. First, it describes the trade-offs in terms of performance, computational requirements and memory consumption of different implementations for the dimensionality reduction and the classification of heartbeats. Second, it presents different optimizations to transform neuro-fuzzy classifiers, allowing their implementation on resource-constrained WBSNs.

Experimental results show that a neuro-fuzzy classifier based on random projections can successfully discard up to 94.9% of the normal heartbeats, while misclassifying only 5% of two different types of abnormal beats. Moreover, a real-world system featuring early heartbeat classification is able to save up to 60% of computational time and 63% in wireless transmission with respect to state-of-the-art approaches even if the proportion of abnormalities is relatively high.

## Figures and Tables

**Table 1.** Composition of the sets of heartbeats employed in the different experiments.

	**N**	**V**	**L**	**Total**
***MIT-BIH Arrhythmia Database***	74,064	6608	8032	88,704
***train_set_1***	150	150	150	450
***train_set_2*** (3 folds)	55,548	4956	6024	66,528
***test_set*** (fold size)	18,516	1652	2008	22,176

**Table 2.** Average Normal Discard Rate (NDR) in the 4-fold cross validation process for a fixed minimum Abnormal Recognition Rate (ARR) of 95%.

**Dimensionality Reduction**	**NFC Coefficients**	

	**8**	**16**
**FPD**	70.25%	-
**RP**	94.80%	94.92%
**RP + FPD**	-	94.19%
**PCA**	88.15%	90.92%
**PCA + FPD**	-	90.04%

**Table 3.** Duty cycle (%) of the sub-systems identified in Figure 6. Tests performed on the IcyFlex WBSN running at 6 MHz.

**Dimensionality Reduction**	**Sub-system**			

	**(1)**	**(2)**	**(3)**	**(4)**
**FPD**	0.63	17.63		54.59
**RP (16)**	1.34	18.34		33.52
**RP (8) + FPD**	1.32	18.32	83.01	34.46
**PCA (16)**	1.24	18.24		34.99
**PCA (8) + FPD**	1.33	18.32		36.45

**Table 4.** Memory footprint (kB) of the sub-systems identified in Figure 6. Tests performed on the IcyFlex WBSN running at 6 MHz.

**Dimensionality Reduction**	**Sub-system**			

	**(1)**	**(2)**	**(3)**	**(4)**
**FPD**	7.75	36.43		82.82
**RP (16)**	3.04	31.72		78.11
**RP (8) + FPD**	8.58	37.25	46.39	83.65
**PCA (16)**	5.33	34.01		80.40
**PCA (8) + FPD**	10.87	39.55		85.94

**Table 5.** Classification performance and number of parameters for different classifiers.

**Classifier**	**NDR**	**ARR**	**Required operations**
**SVM-linear**	93.4%	90.14%	Linear combination of the feature vector (16 elements)
**SVM-gaussian**	96.08%	96.93%	One norm and one gaussian function per SV (210 in total), and their linear combination
**LDA**	93.68%	90.47%	Linear combination of the feature vector (16 elements)
**NFC**	94.19%	96.1%	One gaussian function for each class-feature pair (48 in total) and their product aggregation
